# Chylomicron retention disease caused by a new pathogenic variant in sar1b protein: a rare case report from Syria

**DOI:** 10.1186/s12887-021-02897-5

**Published:** 2021-10-11

**Authors:** Leen Jamel Doya, Lava Mohammad, Razan Omran, Alexander Ali Ibrahim, Nizar Yousef, Ali Ibrahim, Mohammad Adib Houreih

**Affiliations:** 1 Department of Pediatrics, Tishreen University Hospital, Lattakia, Syria; 2 Department of Internal Medicine, Tishreen University Hospital, Lattakia, Syria; 3 Faculty of Medicine, Tishreen University Hospital, Lattakia, Syria; 4 Faculty of Pharmacy, Al-Sham Private University, Lattakia, Syria

**Keywords:** Anderson’s disease, Chylomicron retention disease, Failure to thrive, Fat-soluble vitamin deficiency, Steatorrhea

## Abstract

**Background:**

Chylomicron retention disease (Anderson disease) is a result for variant of the SAR1B gene. It is a rare autosomal recessive hereditary disorder with most incidence in infant. It is characterized by lipid malabsorption syndrome with fatty, chronic diarrhea, and growth retardation.

**Case presentation:**

We report a case of a 19-month Syrian boy who presented with vomiting, growth failure, and chronic, fatty diarrhea. Upper gastrointestinal endoscopy showed whitish appearing duodenal mucosa and small intestinal biopsies revealed steatosis of enterocytes. Genetic testing confirmed chylomicron retention disease with the first description of variant located in the fourth helix of *sar1b* protein. The patient is treated with nutritional supplements and fat-soluble vitamin supplementation resulting in significant improvement.

**Conclusion:**

Early endoscopy is recommended in infants with persistent vomiting and failure to thrive due to high suspicion for a disorder of hypocholesterolemia. Early diagnosis and treatment are essential to avoid serious clinical complications, especially neurological impairment.

## Background

The first clinical history of chylomicron retention disease (CRD) was reported in 1961 by Anderson of a 7-month-old child with persistent neonatal diarrhea. Since then, it has been called Anderson’s disease (AD) [1]. CRD is an autosomal recessive disorder with a prevalence of less than one per million individuals [2]. Since 2003, the *SAR1B* gene was identified as responsible for CRD [3]. To date, about 20 variants have been reported in roughly 60 patients. Information concerning the *SAR1B* genes in the other patients has not been published [2]. Gastrointestinal symptoms as diarrhea, vomiting, abdominal distention that lead to failure to thrive (FTT) are the most prominent in CRD patients that appear at the beginning of life. The absence of characteristic signs and symptoms of CRD often delays diagnosis [4]. Hepatomegaly with elevated liver enzymes may manifest in 20% of CRD patients [5]. A delayed-diagnosis CRD patients should be investigated for the presence of extra-gastrointestinal symptoms as cardiomyopathy, ophthalmic, muscular (cramps, muscular pain), or neurological manifestations (areflexia, ataxia, sensory neuropathy) [6]. Herein we report a case of CRD caused by a new pathogenic variant in sar1b protein in a 19 months old male with steatorrhea, vomiting, and abdominal distension.

## Case presentation

A 19-month-old Syrian boy was referred to our clinic for steatorrhea, vomiting, and abdominal distension. He was born a full-term, normal pregnancy without any complications after birth. The birth weight was 4 kg. The newborn screening test was normal. Since early infancy, he suffered from feeding intolerance and diarrhea described 5 times per day; oily, bulky, and foul-smelling without vomiting or bilious emesis. He was on breast and bottle feeding. By the age of 6 months, there was an obvious failure to weight gain and the family started seeking medical advice. Investigations were performed which excluded primary cystic fibrosis, alpha 1 antitrypsin, Metabolic disease (lipid metabolism disease), thyroid diseases, adrenal insufficiency, diabetes mellitus, and celiac disease. There were fatty drops in the stool examination. A diagnosis of non-specific malabsorption was made and the patient was treated with multivitamins without any improvement in general condition. The family started feeding him PediaSure® formula milk - only by chance- from around 10 months of age with partial improvement in his symptoms and observed weight gain. His development was normal for his age. The child underwent all the compulsory immunizations for his age. There was no relationship between the parents and the family history was noncontributory.

**Fig. 1:**
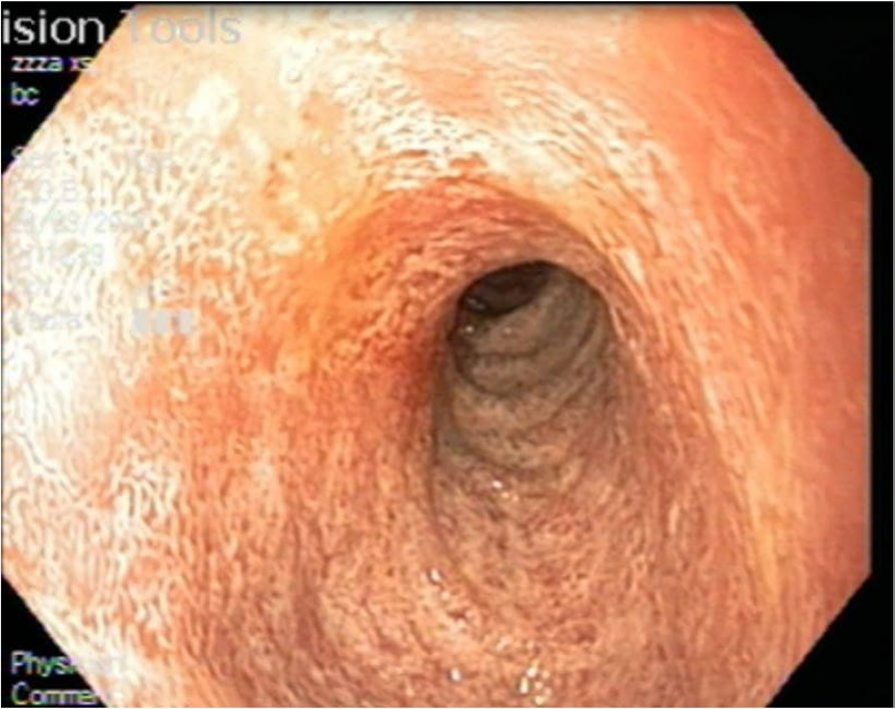
Upper endoscopy reveals a white duodenal mucosa in CRD

**Fig. 2:**
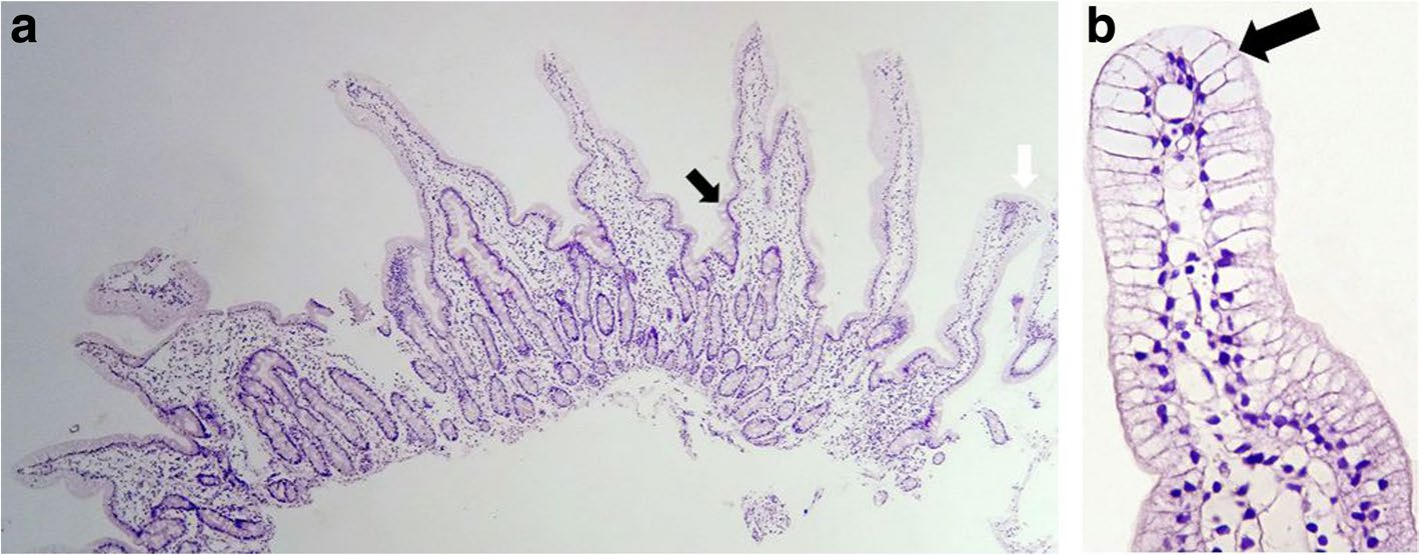
**a**, **b** Histology of the duodenum biopsy. Panel **A** photomicrograph of hematoxylin-eosin staining showing fat-laden as distribution of vacuolization of enterocytes with well-preserved villous structure: fat-filled enterocytes (black arrow) in the upper part of the villus are associated with normal enterocytes in the crypts (white arrow)

On physical examination, his body weight was 9 kg (− 3 SD), the length was 75 cm (− 3 SD), he was vitally stable and generally well. The abdomen was distended but soft, with no masses or organomegaly. The neurological assessment was normal. The patient underwent multiple investigations. Complete blood count (CBC), C- reactive protein (CRP), blood film, blood gases, renal and liver function, electrolytes, glucose, bilirubin, ammonia, amylase, lipase, and urine were normal (Table 1). Stool microscopy revealed fat globules. Abdominal ultrasound showed revealed intestinal wall thickening with normal findings (Liver steatosis was not observed). An ophthalmologic (funduscopic examination), cardiac assessment (echocardiogram) was normal. We performed an Esophagogastroduodenoscopy (EGD) that showed a milky white “snow-storm” appearance of the duodenal mucosa (Fig. 1) and the histological biopsies from the duodenum showed fat-laden vacuolization of enterocytes without villous atrophy (Fig. 2a, b). That suggested lipid trafficking disorder. The Differential Diagnosis included abetalipoproteinemia (ABL), hypobetalipoproteinemia (HBL), or CRD. Therefore, more specialized additional investigations were performed including total cholesterol (TC), Low-Density lipoprotein cholesterol (LDL-C), and total lipid which were markedly reduced; high-Density lipoprotein cholesterol (HDL-C) was borderline low with normal very-low-density lipoprotein (VLDL) and Triglycerides (TG). Total Creatine kinase (CK) was elevated. Vitamin A, D levels were borderline low with low vitamin E levels (Table 2). Lipoprotein electrophoresis of postprandial plasma did not show the presence of chylomicrons. Immunoblot testing showed a decrease in apolipoprotein A1 and apolipoprotein B.

**Table 1:** The laboratory data of the case

Test	Result	Normal range	Test	Result	Normal range
**WBC (10**^**3**^**/μl)**	9.3	6.2- 17	**K (mmol/L)**	4.5	3-4.5
**Neutrophils (%)**	70	40-60	**Na (mmol/L)**	135	135-145
**Lymphocyte (%)**	20	20-40	**ALT (U/L)**	20	7–55
**Hb (g/dl)**	8.7	11-13	**AST(U/L)**	15	5- 40
**MCV(fl)**	60	70-85	**Glucose (mg/dL)**	80	70 -100
**PLT (10**^**3**^**/μl)**	522	150-450	**Amylase (U/l)**	100	70-235
**CRP (mg/dl)**	3	< 5	**Lipase (U/l)**	50	0-200
**Urea (mg/dl)**	30	15-36	**TB (mg/dL)**	0.5	0.2-0.8
**Creatinine (mmol/L)**	0.5	0.5-1.3	**Ammonia (mcg/dl)**	50	25-94

*WBC* White blood cell, *HB* Hemoglobin, *MCV* Mean corpuscular volume, *RDW* Red Cell Distribution Width, *PLT* platelets, *CRP* C-reactive protein, *ALT* Alanine aminotransferase, *AST* Aspartate Aminotransferase, *Na* sodium, *K* potassium

**Table 2:** Pretreatment and post-treatment laboratory findings

Test	Pretreatment	Post-treatment	Normal range
**TC (mg/dl)**	57↓	150	125-200
**TG (mg/dl)**	86↔	90	40-150
**LDL-C (mg/dl)**	29↓	65	50-100
**HDL-C (mg/dl)**	45↔	60	> 45
**VLDL (mg/dl)**	17↔	20	10-40
**Total lipid (mg/dl)**	304↓	500	450-900
**CK (U/l)**	242↑	124	Up to 190
**Vit E (mg/ L)**	1.9↓	9.5	9-21
**25 OH D3 (ng/ l)**	25.1↔	30	25-80
**Vit A (mg/ L)**	0.3↔	0.5	0.3-0.9

*TC* Total cholesterol, *TG* triglyceride, *LDL-C* Low-density lipoprotein, *HDL-C* High-density lipoprotein, *VLDL* very low-density lipoprotein, *CK* creatine kinase

The clinical and laboratory findings were more consistent with CRD. A blood sample for genetic analysis in directional direct DNA sequencing (AB 3730 DNA analyses equipped with seascape software) of SARB coding exons and exonic-intronic junctions from genomic DNA was obtained. Genotyping identified a homozygous SAR1B gene variant as the deletion of one of the two repeated Glutamine in position 113 or 114. It was the first description of this variant in the fourth helix of *SAR1B* protein *(NM_0161032.: c.[del340-342]; [del340-342], p. [Glu114del]; [Glu114del])*. His parents were heterozygous for the same variant *(NM_0161032.: c.[del340-342]; [=], p. [Glu114del]; [=]).*

The patient was started on the restriction of dietary fats, hydrolyzed formula with a higher medium-chain triglyceride content, some vegetable oils (sun-seed, olive oil, almond oil) in his daily diet in addition to fat-soluble vitamin supplementation (hydrosoluble vitamin E 50 UI/kg/d; vitamin A 15,000 IU/d; vitamin K 15 mg/wk.; vitamin D 1000 UI/kg/d).

**Fig. 3:**
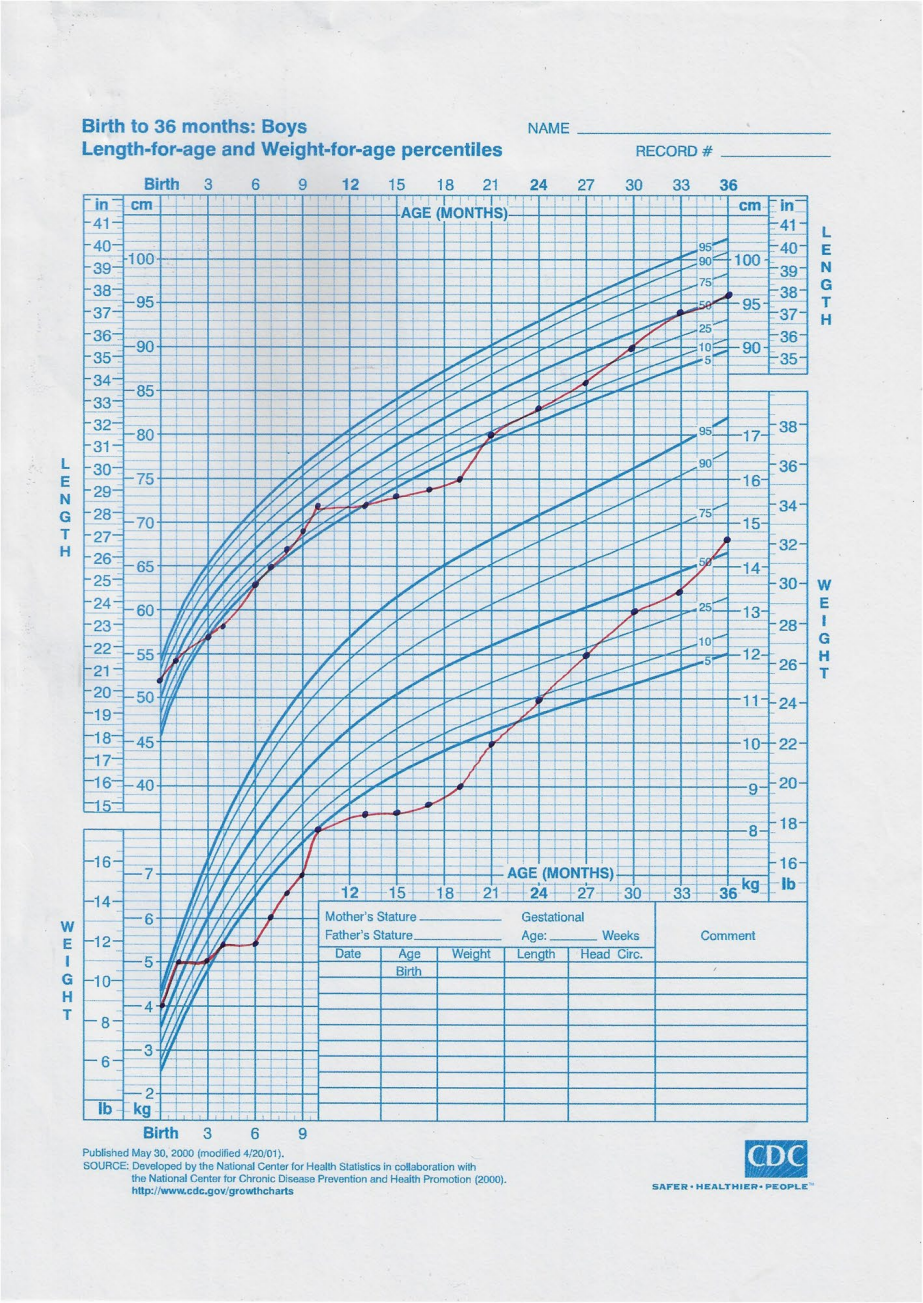
The growth charts of the patient before and after treatment

During follow-up, the child showed catch-up growth in weight–height and a clinical examination were unremarkable with normal laboratory findings (Table 2) (Fig. 3).

## Discussion and conclusion

In the literature review 2018, there were about 62 cases of CRD with described mutations in the *SAR1B* gene [7]. The *SAR1B* gene is essential in the transfer of the chylomicron from the endoplasmic reticulum (ER) to the Golgi apparatus by encoding the Sar1b protein. The Sar1b variant caused the accumulation of pre-chylomicron transport vesicles in the cytoplasm of the enterocytes [3]. As shown in the literature clinical case reports, diarrhea had been reported in all CRD patients, except one who has been informed by M.Woods et al. 2018 of a 5-month-old French Canadian male who had only vomiting and severe failure to thrive [8]. CRD is associated with fat-soluble vitamin deficiency (A, D, E, K) that can cause serious complications (Table 3). Early diagnosis of vitamins helps in reducing the long-term complications that may not be inversed fully with supplementation [9].

**Table 3:** Fat-soluble vitamin deficiency in CRD patients

Deficiency	Symptoms
**Vitamin A**	Visual and skin changes
**Vitamin D**	Bone abnormalities (delayed growth, poor bone mineralization)
**Vitamin E**	neurological abnormalities (sensory or motor neuropathy)
**Vitamin K**	hemorrhagic disorders and Blood clotting problems

Our patient presented with nonspecific signs of CRD (vomiting, growth failure, and chronic fatty diarrhea). Laboratory and EGD findings were directed to CRD. The diagnosis was made based on the genetic analysis. Because of this possibility of accompanying systemic demonstrations, the patient was evaluated by Neurology, Ophthalmology, and Cardiology that were normal.

The diagnosis is made by laboratory profiles (Table 4) and supported by the upper endoscopy that reveals a milky white “snow-storm” appearance of duodenal mucosa with normal esophageal and normal gastric mucosa. To increase endoscopic sensitivity, a TG-rich diet started 3 days before the exam to improve the fat loading of enterocytes [5].

**Table 4:** The laboratory profiles and treatment of CRD

The laboratory profiles	
**Hypocholesterolemia:**	
↓ TC, ↓ HDL-c, ↓LDL-c**,** normal TG	
acanthocytosis, ↑CK	
↓ fat-soluble vitamins (A, D, E, K)	
↓essential fatty acids (EFA)	
**Treatment**	
exclusion of long-chain fatty acids from the diet	
**supplementation with fat-soluble vitamins:**	
**hydrosoluble vitamin E:** 50 UI/kg/d	
**vitamin A:** 15,000 IU/d	
**vitamin K:** 15 mg/wk.	
**vitamin D:** 800-1200 UI/kg/d; 100,000 IU/2 months (≤5-yr), 600,000 IU/2 months (> 5-yr)	

*TC* total cholesterol, *HDL-c* high-density lipoprotein cholesterol, *LDL-c* low-density lipoprotein cholesterol, *TG* triglycerides, *CK* creatine kinase, *EFA* essential fatty acids

Histology shows normal villous to crypt ratio and multi-vacuolated enterocytes. CRD may be confused with celiac disease in some patients who demonstrate mild atrophic villi without identifying the lipid vacuoles. Furthermore, the percentage of lipid-laden villi, the area of ​​affected villi, and their extension can vary substantially in the same patient and among different patients. In most cases, the vacuolization is seen only in the upper one-third of the villi as in our case. Finally, the last step for diagnosis, which is the gold standard, is the identification of the *SAR1B* gene variant [5].

The management of CRD focuses on two important points. The first is the exclusion of long-chain fatty acids from the diet, to decrease the fat load inside the enterocytes, and to promote absorption. In very young children, milk preparations with medium-chain TG may improve diarrhea and correct malnutrition within a few days but tolerance can be a problem. In older children, a regimen low in long-chain fatty acids is usually sufficient to decrease symptoms. The second is supplementation with fat-soluble vitamins to prevent vitamin deficiency syndromes (Table 4) [10]. Although by chance our patient started on PediaSure® formula milk, he had consumed long-chain fatty acids from other complex nutrients. The parents are advised to adhere to both the special diet and the supplementary fat-soluble vitamins and the baby responded well.

This case describes a SAR1B gene mutation never previously described. Even though CRD is rare, pediatricians should have a high index of suspicion of this disease in patients with nonspecific symptoms like vomiting, growth failure, and chronic, fatty diarrhea. Early diagnosis, dietary counseling, and supplementation with fat-soluble vitamins will lead to good outcomes and avoidance of serious clinical sequelae, especially neurological impairment.

## Data Availability

All data generated or analyzed during this study are included in this published article.

## Abbreviations

ABL: Abetalipoproteinemia
AD: Anderson’s disease
ALT: Alanine aminotransferase
AST: Aspartate Aminotransferase
CBC: Complete blood count
CRD: Chylomicron retention disease
CK: Creatine kinase
CRP: C- reactive protein
EFA: Essential fatty acids
EGD: Esophagogastroduodenoscopy
ER: Endoplasmic reticulum
FTT: Failure to thrive
HB: Hemoglobin
HBL: Hypobetalipoproteinemia
HDL-C: High-density lipoprotein
K: Potassium
LDL-C: Low-density lipoprotein
MCV: Mean corpuscular volume
Na: Sodium
PLT: Platelets
RDW: Red Cell Distribution Width
TC: Total cholesterol
TG: Triglyceride
VLDL: Very low-density lipoprotein
WBC: White blood cell
